# Distribution of IL28B Genotypes in Iranian Patients with Chronic Hepatitis C and Healthy Individuals

**DOI:** 10.5812/hepatmon.8387

**Published:** 2012-12-22

**Authors:** Heidar Sharafi, Ali Pouryasin, Seyed Moayed Alavian, Bita Behnava, Maryam Keshvari, Shima Salimi, Leila Mehrnoush, Ahmad Fatemi

**Affiliations:** 1 Armin Pathobiology Laboratory, Tehran, Iran; 2 Tehran Hepatitis Cohort (THC) Study Center, Tehran, Iran; 3 Department of Genetics, Islamic Azad University of Arsanjan, Arsanjan, Iran; 4 Baqiyatallah Research Center for Gastroenterology and Liver Disease, Baqiyatallah University of Medical Sciences, Tehran, Iran; 5 Iranian Blood Transfusion Organization, Tehran, Iran; 6 Department of Hematology, Tehran University of Medical Sciences, Tehran, Iran

**Keywords:** Polymorphism, Genetic, Hepatitis C, IL28B Genotype, Human, Persian Gulf, Iran

## Abstract

**Background:**

IL28B polymorphism is recognized as one of the most prominent predictors of hepatitis C spontaneous and treatment-induced clearance. Interestingly, the favorable genotypes of IL28B are found to be more frequent in Asian ethnicity than Caucasian and African populations, respectively. A few studies reported that there is a mysterious association between the IL28B polymorphism and the hepatitis C virus (HCV) genotype in patients with chronic hepatitis C but they did not give any reason for this phenomenon.

**Objectives:**

The foremost purpose of this study was to compare the distribution of IL28B genotypes between Iranian healthy individuals and patients with chronic hepatitis C.

**Patients and Methods:**

In this study, 921 patients with chronic hepatitis C and 142 healthy individuals were included. The IL28B rs12979860 and rs8099917 polymorphisms were genotyped using the polymerase chain reaction-restriction fragment length polymorphism (PCR-RFLP) method.

**Results:**

The frequency of IL28B rs12979860 CC, CT, and TT genotypes in chronic hepatitis C patients was 38%, 48.8%, and 13.2% and in healthy individuals was 43.7%, 48.6%, and 7.7%. Also, the frequency of IL28B rs8099917 TT, GT, and GG genotypes in chronic hepatitis C patients was 58.3%, 37.1%, and 4.6% and in healthy individuals was 64.1%, 32.4% and 3.5%. The differences in the distribution of IL28B rs12979860 and rs8099917 genotypes between patients with chronic hepatitis C and healthy individuals were not statistically significant. When we compared the distribution of IL28B genotypes between the healthy group and the HCV infected patients by HCV genotype, we found 9.8% higher frequency of rs12979860 CC genotype in the healthy individuals than HCV genotype 1 infected patients (P = 0.03) however there was no significant difference in the distribution of rs12979860 genotypes between the healthy and HCV genotype 3 infected groups (P = 0.46).

**Conclusions:**

It seems that the impact of IL28B polymorphism on the spontaneous clearance of HCV genotype 1 is more prominent than HCV genotype 3 which results in the observation of higher rs12979860 C allele frequency in chronic hepatitis C patients with HCV genotype 3 than HCV genotype 1.

## 1. Background

Hepatitis C infection with a worldwide 3% prevalence is a major health concern (1). Spontaneous clearance occurs in 20%-25% of patients with acute hepatitis C infection and as a result 70%-75% of cases progress to chronic hepatitis C (2). Chronic hepatitis C is one of the major causes of cirrhosis and hepatocellular carcinoma, globally (3). The current standard of care for the treatment of patients with hepatitis C is Pegylated interferon alpha (Peg-IFN) in combination with ribavirin (RBV). Treatment with this regimen results in 40%-50% eradication of infection in hepatitis C virus (HCV) genotype 1 infected patients and 70%-80% in HCV genotype 3 infected patients (4). Recently, the single nucleotide polymorphisms (SNPs) in the genomic region of IL28B gene have been found to be highly associated with spontaneous clearance and treatment related clearance of hepatitis C infection (5-9). Among IL28B polymorphisms which are associated with hepatitis C treatment response, two polymorphisms including rs12979860 and rs8099917 were studied more than others (10). The IL28B rs12979860 CC and rs8099917 TT genotypes are associated with a higher probability of spontaneous clearance and treatment-induced viral clearance than IL28B rs12979860 T allele carriers and rs8099917 G allele carriers. Although the distribution of IL28B genotypes is varied in various races, which can explain the variation in antiviral response rates by ethnicity, association of IL28B SNPs and treatment-related response and spontaneous clearance of hepatitis C was virtually independent of the ethnicity of study population (5, 9) and as a result, numerous studies worldwide have reported the strong impact of the IL28B polymorphisms on the natural history and treatment-related response of hepatitis C. Another interesting finding in the recent studies was the mysterious association between the HCV genotype and IL28B polymorphisms in which the frequency of IL28B favorable alleles were higher in the HCV genotype 2 and 3 infected patients than HCV genotype 1 infected patients (11, 12).

## 2. Objectives

Objectives of the current study include: 1) Determination of the distribution of IL28B rs12979860 and rs8099917 genotypes in Iranian patients with chronic hepatitis C, 2) Determination of the distribution of IL28B rs12979860 and rs8099917 genotypes in Iranian healthy individuals, 3) Comparison of the distribution of IL28B rs12979860 and rs8099917 genotypes between Iranian healthy individuals and patients with chronic hepatitis C

## 3. Patients and Methods

### 3.1. Study Population

In this cross-sectional study, 921 Iranian patients with chronic hepatitis C infection, whom were referred to Tehran Hepatitis Center – clinical department of Baqiyatallah Research Center for Gastroenterology and Liver Disease (BRCGL) were included. Patients who had cleared the infection spontaneously were excluded from the study. All of the patients were anti-HCV antibody positive and had positive Plasma HCV RNA by RT-PCR for more than 6 months. Furthermore a group of 142 Iranian healthy, anti-HCV antibody and HBsAg negative, individuals were included as the control group. All individuals in both groups were Caucasian. 

### 3.2. Laboratory Assessment of Host and Viral Factors

The HCV genotype of patients with chronic hepatitis C were assessed in different medical diagnostic laboratories using valid methods such as DNA sequencing, and methods using genotype specific primers and polymerase chain reaction-restriction fragment length polymorphism (PCR-RFLP). The IL28B rs12979860 and rs8099917 SNPs were genotyped by the PCR-RFLP method as formerly described (13) Briefly, genomic DNA of hepatitis C infected patients and healthy individuals were extracted from their peripheral blood using QIAamp® DNA Blood Mini Kit (Qiagen, Hilden, Germany) according to the manufacturer’s instructions. 100-300 ng of genomic DNA was amplified using 10 pmol of primers 5’-GCGGAAGGAGCAGTTGCGCT-3’ and 5’-GGGGCTTTGCTGGGGGAGTG-3’ in the PCR amplification for genotyping of IL28B rs12979860 SNP and 100-300 ng of genomic DNA was amplified using 10 pmol of primers 5’-CCCACTTCTGGAACAAATCGTCCC-3’ and 5’-TCTCCTCCCCAAGTCAGGCAACC-3’ in a separate PCR for genotyping of IL28B rs8099917 SNP. The PCR was performed using Accupower® PCR PreMix (Bioneer, South Korea) with a 20 µl reaction tube type. The temperature profile which was used for the PCR amplification of both SNPs consisted of a 94°C for 5 minutes, followed by 35 cycles of 94°C for 20 seconds, 66°C for 20 seconds and 72°C for 20 seconds, followed by a 72°C for 5 minutes. For the RFLP assay, the PCR amplicon containing the rs12979860 SNP was digested with 10 units of Bsh1236I (BstUI) restriction endonuclease (Fermentas, Vilnius, Lithuania) and the PCR amplicon containing the rs8099917 SNP was digested with 10 units of BseMI (BsrDI) restriction endonuclease (Fermentas, Vilnius, Lithuania) for at least one hour. The digested PCR products were separated on 3% agarose gel. The rs12979860 CC genotype produced two 196 and 45 bp fragments, the CT genotype produced three 241, 196, and 45 bp fragments and the TT genotype produced a 241 bp fragment. The rs8099917 TT genotype produced a 552 bp fragment, the GT genotype produced three 552, 322 and 230 bp fragments and the GG genotype produced two 322 and 230 bp fragments. 

### 3.3. Statistical Analysis

Statistical differences between groups were determined using Chi-square test. P ≤0.05 was considered statistically significant. Statistical analysis was performed using SPSS version 17.0 and OpenEpi version 2.3.

## 4. Results

### 4.1. Patient’s Characteristics

The mean ± SD of age in the group of the patients with chronic hepatitis C and in the group of the healthy individuals were 34.5 ± 12 and 46.7 ± 11.6 year old, respectively. The male to female ratio in the patients with chronic hepatitis C was 779/142 and in the healthy individuals was 55/87. 

### 4.2. Distribution of HCV Genotypes in the Patients with Chronic Hepatitis C

Among 921 patients with chronic hepatitis C, 493 (53.5%) were infected with HCV genotype 1a, 306 (33.2%) with HCV genotype 3a, 94 (10.2%) with HCV genotype 1b, 8 (0.9%) with HCV genotype 4, 2 (0.2%) with HCV genotype 2, 2 (0.2%) with HCV genotype 3k and 16 were infected with multiple genotypes of HCV. 

### 4.3. Distribution of IL28B Genotypes in Patients with chronic hepatitis C and Healthy Individuals

Distribution of IL28B rs12979860 and rs8099917 genotypes were in Hardy-Weinberg equilibrium in both patients with chronic hepatitis C and healthy individuals. Among patients with chronic hepatitis C, the distribution of IL28B rs12979860 genotypes was as the following: 350 (38%) were CC, 449 (48.8%) were CT and 122 (13.2%) were TT. In addition, the distribution of IL28B rs8099917 genotypes among the patients with chronic hepatitis C was as the following: 537 (58.3%) were TT, 342 (37.1%) were GT and 42 (4.6%) were GG. Among the healthy individuals, the distribution of IL28B rs12979860 genotypes was as the following: 62 (43.7%) were CC, 69 (48.6%) were CT and 11 (7.7%) were TT. Also, the distribution of IL28B rs8099917 genotypes among the healthy individuals was as the following: 91 (64.1%) were TT, 46 (32.4%) were GT and 5 (3.5%) were GG. The differences in the distribution of IL28B genotypes and alleles between patients with chronic hepatitis C and healthy individuals were not statistically significant (Figure 1) IL28B rs12979869 and rs8099917 genotypes and alleles in HCV genotype 3 infected patients were the same as the healthy individuals.

Distribution of IL28B Genotypes and Alleles in the Healthy Individuals and Patients with Chronic Hepatitis C

**Figure 1A):**
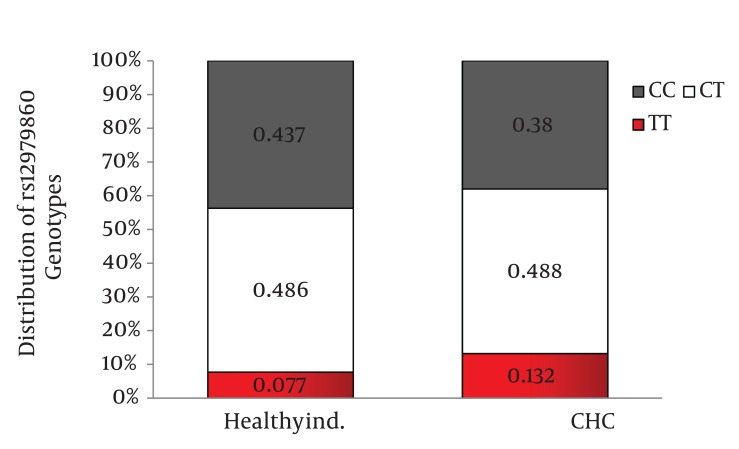
Distribution of rs12979860 Genotypes

**Figure 1B):**
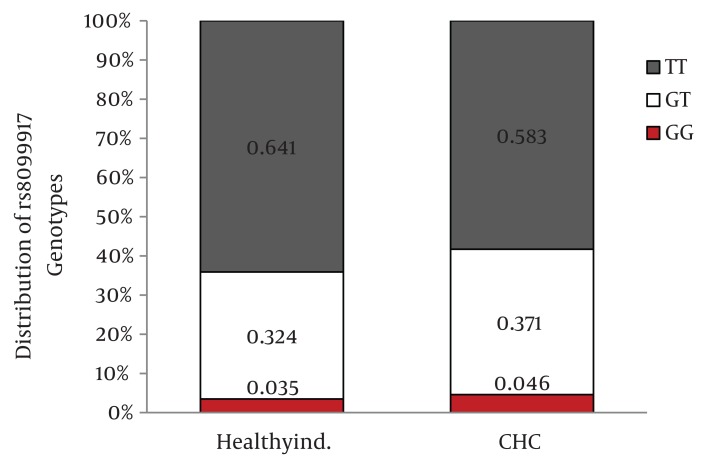
Distribution of rs8099917 Genotypes

**Figure 1C):**
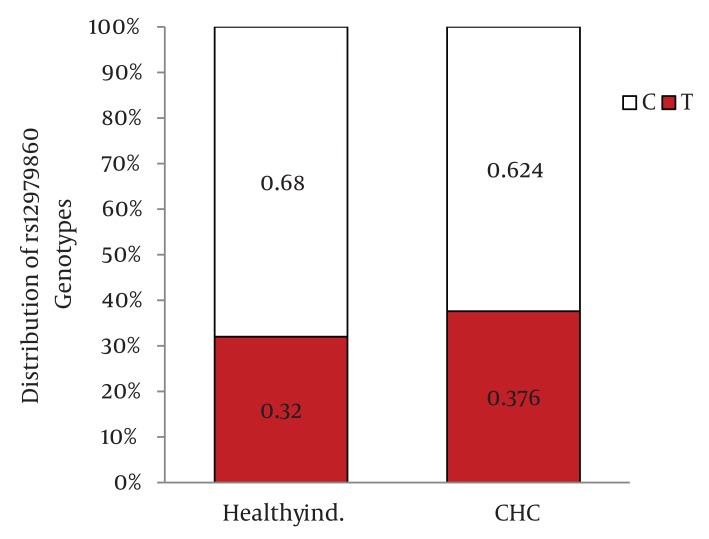
Distribution of rs12979860 alleles

**Figure 1D):**
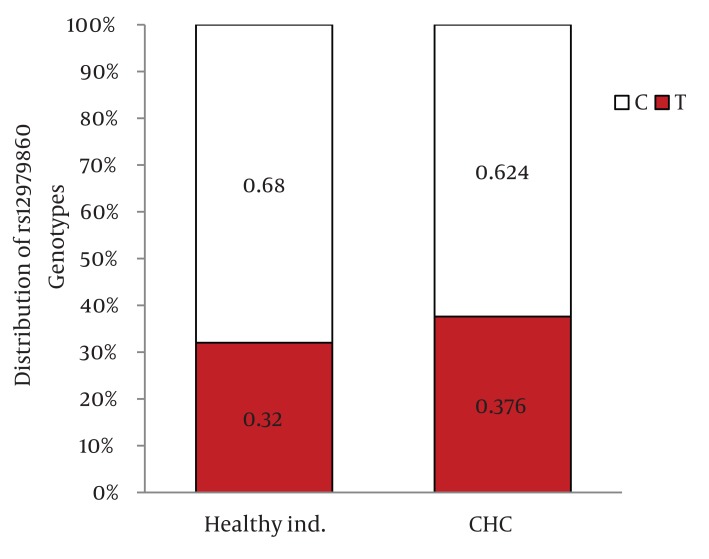
Distribution of rs8099917 alleles CHC, Chronic Hepatitis C; Healthy ind., Healthy Individuals

When we compared the distribution of IL28B rs12979860 genotypes and alleles between HCV genotype 1 infected patients and healthy individuals, we found 9.8% higher frequency of rs12979860 CC genotype in the healthy individuals than HCV genotype 1 infected patients (P = 0.03)(Figure 2A) and 8.3% higher frequency of rs12979860 C allele in healthy individuals than HCV genotype 1 infected patients (P = 0.01) (Figure 2C). We did not find a significant difference in the frequency of rs8099917 TT genotype between HCV genotype 1 infected patients and healthy individuals (P = 0.13) (Figure 2B) but there was a significant difference in the frequency of rs8099917 T allele between healthy individuals and HCV genotype 1 infected patients (P = 0.05) (Figure 2D). The differences in the distribution of rs12979860 and rs8099917 genotypes and alleles between HCV genotype 1 and HCV genotype 3 infected patients were statistically significant (Figure 2) . Another interesting finding was 9.7% higher frequency of rs12979860 T allele in HCV genotype 1b infected patients than HCV genotype 1a infected patients which was statistically significant (P = 0.01).

Distribution of IL28B Genotypes and Alleles in the Healthy Individuals and Patients with Chronic Hepatitis C by HCV Genotype

**Figure 2A):**
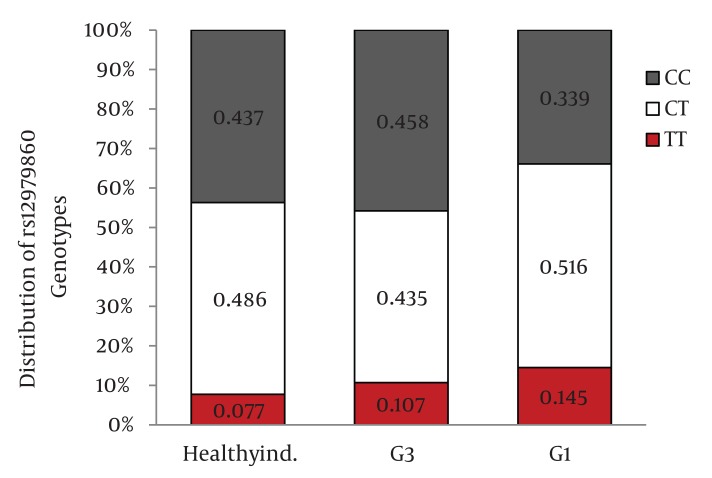
Distribution of rs12979860 Genotypes

**Figure 2B):**
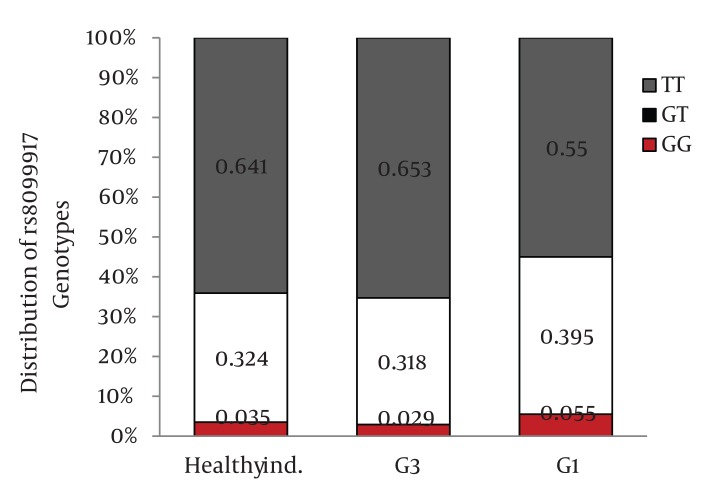
Distribution of rs8099917 Genotypes

**Figure 2C):**
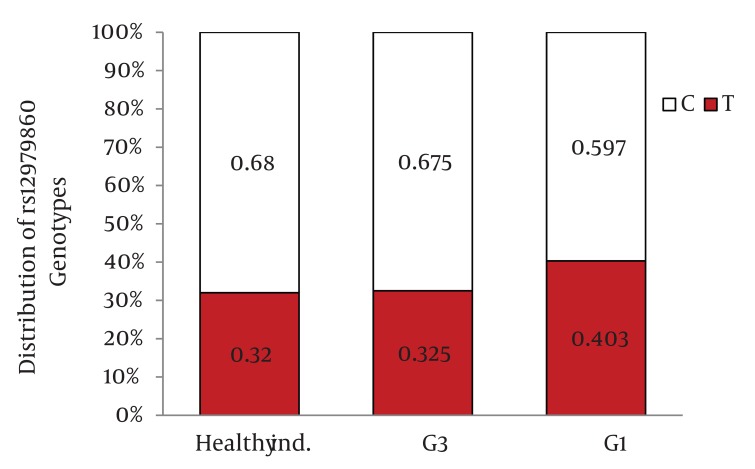
Distribution of rs12979860 alleles

**Figure 2D):**
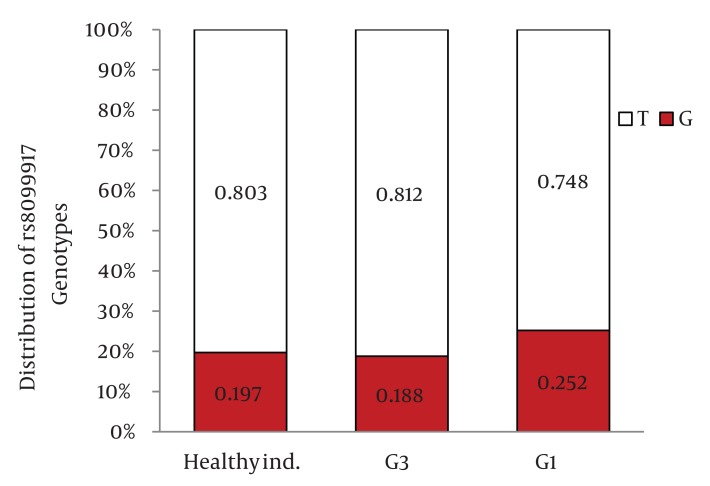
Distribution of rs8099917 alleles G1, HCV Genotype 1 Infected Patients; G3, HCV Genotype 3 Infected Patients; Healthy ind., Healthy Individuals

## 5. Discussion

In the present study in Iran, located in the Middle East region with Caucasian ethnicity, the most common IL28B rs12979860 genotype in chronic hepatitis C patients and healthy individuals was CT followed by CC and TT. Furthermore, the most prevalent rs8099917 genotype was TT, followed by GT and GG. The most prevalent IL28B rs12979860 and rs8099917 alleles were C and T, consequently. The former studies in Caucasian patients in Europe, United State, and Australia showed that the most common rs12979860 genotype was CT and then CC and TT (6, 14). The most prevalent rs8099917 genotype was TT followed by GT and GG, consequently. There has been little evidence about the prevalence of IL28B genotypes in the Middle East countries in the literature. In this study, the prevalence of rs12979860 and rs8099917 genotypes in Iran was compared with other Caucasian patients. As we know the rs12979860 C allele is the favorable allele for the spontaneous clearance of HCV and the prediction of response rate to Peg-IFN and RBV in patients infected with genotype 1 or 4. In this study there was no difference in the prevalence of rs12979860 and rs8099917 genotypes between total chronic hepatitis C patients and the healthy group, but a significant difference was seen if the healthy group was compared with patients who were infected with genotype 1. On the other hand, the prevalence of rs12979860 CC in patients infected with HCV genotypes 1 was lower than the healthy group. Furthermore, the frequency of rs12979860 C allele in patients with HCV genotype 1 was lower than the healthy control and patients infected with genotype 3. However, the frequency of rs12979860 C allele in HCV infected patients with genotype 3 was the same as the healthy control. Similarly, two recent studied by Montes-Cano et al. and Falleti et al. have reported that the prevalence of rs12979860 CC genotype in patients infected with HCV genotype 1 was lower than the healthy group and also lower than patients with HCV genotype 3 (11, 12). But in patients with genotype 2 or 3 there was no difference in the prevalence of IL28B genotypes between them and healthy control group. McCarthy et al. have reported that the prevalence of rs12979860 CC was higher in patients infected with HCV genotype 3 than genotype 1 (15). Two previous studies in HCV infected patients with genotype 1 and 4 reported that the spontaneous clearance of HCV was higher in hepatitis C patients with rs12979860 CC genotype than the other rs12979860 genotypes (16, 17). Likewise, in a study in patients co-infected with HIV, spontaneous clearance occurred only in HCV genotype 1 patients (18). As a result, it seems that in the acute phase of HCV infection with viral genotypes 1 or 4, those who have IL28B rs12979860 CC genotype clear the virus better than rs12979860 T allele carriers, so in the chronic hepatitis C with HCV genotype 1, rs12979860 CC genotype is seen in lower frequency than the healthy group. On the other hand, IL28B polymorphisms probably have an insignificant role in the spontaneous clearance of HCV genotype 3 infected patients which results in the observation of the same distribution of IL28B genotypes in the HCV genotype 3 infected patients and healthy individuals. Similar results were seen in chronic hepatitis C patients who have been treated with combination of Peg-IFN and RBV. It has been shown that in HCV infected patients with genotypes 1 or 4, those with rs12979860 CC genotype have higher response rate to combination therapy than T allele carriers (19) while, a recent meta-analysis showed a poor effect of IL28B rs12979860 SNP on response rate to combination therapy in patients infected with genotype 3 (20). In conclusion, these data suggested that rs12979860 SNP may act differently in the clearance process of different HCV genotypes and it can predict the spontaneous clearance rate of HCV in patients infected with genotypes 1. We suggest more studies especially in the acute phase of hepatitis C in different viral genotypes that can better show the interaction of HCV genotype and IL28B polymorphisms.
